# Piperlongumine reverses doxorubicin resistance through the PI3K/Akt signaling pathway in K562/A02 human leukemia cells

**DOI:** 10.3892/etm.2015.2254

**Published:** 2015-02-03

**Authors:** QINGWEI KANG, SHU YAN

**Affiliations:** Pharmacy Department, Tianjin Nankai Hospital, Tianjin 300100, P.R. China

**Keywords:** piperlongumine, human leukemia, doxorubicin resistance

## Abstract

Drug resistance is an important obstacle to human leukemia therapeutics. Piperlongumine has previously demonstrated the ability to suppress certain human tumor processes; however, the ability of piperlongumine to reverse the drug resistance of human leukemia and its mechanism of action have not yet been clearly elucidated. In this study, the doxorubicin resistance reversal effect of piperlongumine on K562/A02 human leukemia cells and the underlying mechanism were investigated. The results indicated that piperlongumine promoted doxorubicin sensitivity, apoptosis, the intracellular accumulation of rhodamine-123, the activities of caspase-3 and -8, and the expression of reactive oxygen species, p53, p27 and p-PTEN. Furthermore, it suppressed the expression of P-glycoprotein, MDR1, MRP1, survivin and p-Akt, and the transcriptional activities of NF-κB and twist, and arrested the cell cycle in the G2/M phase. The results indicate that piperlongumine has the potential to be used as a therapeutic agent for human leukemia.

## Introduction

Leukemia results from abnormal functioning of the hematopoietic tissues in bone marrow due to variations of intracellular DNA molecules. As the overproduction of immature leucocytes interferes with other functions of the bone marrow, the capability of bone marrow to make other blood cells declines inevitably. Leukemia cells can spread to the lymph nodes, spleen, liver, central nervous system and other organs. The incidence and mortality of leukemia as a malignant clonal disorder of the hematopoietic stem cells rank first among all pediatric malignancies (1). The exact causes of leukemia remain under study, but DNA variations in bone marrow stem cells generally cause their deterioration, and these may arise due to exposure to radiation, contact with carcinogens and variations in the genetic materials in other cells (2). In addition, viruses may cause leukemia (3,4).

Leukemia drug resistance denotes the insensitivity or resistance of leukemia cells to chemotherapeutic drugs. When a patient has received several courses of chemotherapy, but the percentage of leukemia cells in the bone marrow does not decline significantly or returns to the pretreatment level soon after a short-term discontinuation, this condition is considered to be leukemia drug resistance. Currently, the development of leukemia cell drug resistance is one of the major causes of leukemia treatment failure (5,6).

Multidrug resistance (MDR) is the main reason for chemotherapeutic failure. A number of studies have confirmed that piperlongumine is able to induce the apoptosis of leukemia cells and inhibit their proliferation (4,7,8). Piperlongumine has also been shown to have an inhibitory effect on tumor proliferation, migration, invasion and angiogenesis by oxidative stress (7,8). Although piperlongumine is thought to be able to regulate cell growth, survival, differentiation, proliferation, migration and other cell processes in addition to its involvement in multiple signal transduction pathways, it is still unknown whether piperlongumine is able to reverse drug resistance in leukemia (9–11).

The present study aimed to investigate the reversal effect of piperlongumine on drug resistance in a human leukemia cell line in order to establish an experimental basis for its clinical applications.

## Materials and methods

### Cell lines and cell culture

The K562 human leukemia cell line was purchased from the Shanghai Institute of Cell Biology, Chinese Academy of Sciences (Shanghai, China), and the doxorubicin-resistant K562/A02 cell line was purchased from the Tianjin Institute of Hematology (Tianjin, China). The cells were cultured at 37°C with 5% CO_2_ in Dulbecco’s modified Eagle’s medium with 10% fetal bovine serum (Sigma Aldrich, St. Louis, MO, USA). Piperlongumine (Sigma Aldrich) was dissolved in DMSO to prepare a stock solution, which was further diluted to the experimental concentrations with serum-free culture medium and filtered immediately.

### MTS assay

A total of 5×10^3^ cells were seeded into each well of a 96-well plate and the cells were incubated for 24 h at 37°C with 5% CO_2_. Then, 0, 2, 5, 10, 20, 50 or 100 μM piperlongumine was added and the cells were cultured for a further 24 h. Fresh culture medium was then added, followed by 20 μl (3-(4,5-dimethylthiazol-2-yl)-5-(3-carboxymethoxyphenyl)-2-(4-sulfophenyl)-2H-tetrazolium) (MTS; Promega, Madison, WI, USA). Following the incubation of the cells with MTS for 4 h, the absorbance at 490 nm was determined using a microplate reader.

The MTS assay was repeated using the same method, with the exception that 0, 2, 5, 10, 20, 50 or 100 μM doxorubicin and 2 or 5 μM piperlongumine were added prior to the 24-h culture instead of the various concentrations of piperlongumine.

### Flow cytometric assay

A total of 5×10^5^ cells were seeded into a 6-well plate and cultured for 24 h. Then, 1 μM doxorubicin with 2 or 5 μM piperlongumine was added and the cells were cultured for a further 24 h. The cells were then collected and incubated with fluorescein isothiocyanate (FITC)-annexin V and propidium iodide (PI; BD Franklin Lakes, NJ, USA) for 15 min in the dark. Following the incubation, the fluorescence intensity of the cells was measured at 488 nm by flow cytometry (BD FACSCalibur, BD) for the analysis of apoptosis.

In further experiments, 5×10^5^ cells were seeded into a 6-well plate and cultured for 24 h. Then, 2 or 5 μM piperlongumine was added and the cells were cultured for a further 24 h. Following the 24-h incubation, several different analyses were conducted. i) The cells were collected, fixed and incubated with PI for 30 min in the dark prior to measurement of the fluorescence intensity at 488 nm by flow cytometry to evaluate the cell cycle; ii) the cells were collected, fixed and incubated with dichlorodihydrofluorescein diacetate (DCFH-DA; Beyotime, Shanghai, China) for 30 min in the dark, and then the fluorescence intensity was measured at 488 nm by flow cytometry to evaluate the production of reactive oxygen species (ROS); iii) the cells were collected and incubated with rhodamine-123 (Rh-123; Beyotime) for 30 min in the dark, and then the fluorescence intensity was measured at 488 nm by flow cytometry to determine the intracellular content of Rh-123; and iv) the cells were collected and incubated with P-glycoprotein-phycoerythrin (P-gp-PE) antibody (Beyotime) for 30 min in the dark, and then the fluorescence intensity was measured at 488 nm by flow cytometry to determine the expression of P-gp.

### Realtime polymerase chain reaction (qPCR) assay

A total of 5×10^5^ cells were seeded into a 6-well plate and cultured for 24 h. Then, 2 or 5 μM piperlongumine was added and the cells were cultured for another 24 h. A total of 20 μM RNA was extracted from these cells using the TRIzol method (Beyotime) and the reaction was completed in a ABI 7500 Real-Time fluorescence quantitative PCR instrument (Applied Biosystems, Foster City, CA, USA). The PCR products were labeled with SYBR-Green I (Takara Biotechnology, Dalian, China). The target genes and reference gene GAPDH in all samples were subjected to fluorescence qPCR to obtain the respective CT values; the relative gene expression differences in each sample were analyzed using 2^−ΔΔCT^ method. The primer sequences were retrieved from the online primer database Primer Bank (http://pga.mgh.harvard.edu/primerbank/), and the primers were synthesized and purified by Takara Biotechnology (Dalian) Co., Ltd. (Dalian, China). The PCR process comprised 30 cycles of 94°C for 1 min, 56°C for 50 sec, and 72°C for 1 min. The genes that were detected and their primer sequences are shown as Table I.

### Western blotting assay

A total of 5×10^5^ cells were seeded into a 6-well plate and cultured for 24 h. Then, 2 or 5 μM piperlongumine was added and the cells were cultured for a further 24 h. The cells were then lysed and 100 μg total proteins were separated using a 12% SDS-PAGE gel. The proteins were transferred onto a polyvinylidene difluoride membrane (Millipore, Billerica, MA, USA) and blocked with 5% non-fat milk overnight at 4°C. The membrane was incubated with primary monoclonal mouse anti-human antibodies (MDR1, 1:800; MRP1, 1:800; p27, 1:800; survivin, 1:800; p53, 1:800; p-Akt, 1:400; p-PTEN, 1:800) and β-actin (1:5,000, Sigma) at 4°C overnight. The membranes were washed with PBST buffer (80 mM Na_2_HPO_4_, 20 mM NAH_2_PO_4_, 100 mM NaCl, 0.05% Tween) and incubated for 1 h with horseradish peroxidase-conjugated anti-rabbit secondary antibody (1:5,000) at room temperature. Blots were visualized using an electrochemiluminescence (ECL; Millipore, Billerica, MA, USA) Western Blotting kit. β-actin acted as the internal control.

### Caspase activity

The activities of caspase-3 and -8 were measured using the Apo-ONE homogeneous caspase-3/7 assay (Beyotime, Shanghai, China) following the manufacturer’s instructions. In each assay, 5×10^3^ cells were seeded into a 96-well plate and cultured for 24 h. Then 2 or 5 μM piperlongumine and the caspase substrate (Z-DEVD)_2_-R110 were added, and culturing was continued for a further 7 h for the detection of caspase-3 activity or 3 h for the detection of caspase-8 activity. Then, the fluorescence intensity was measured at 490 nm using a microplate reader (Multiskan MK3, Hudson, Thermo, NH, USA) to determine the activity of caspase-3/8.

### Assay of the transcriptional activity of NF-κB and twist

A total of 5×10^5^ cells were seeded into a 6-well plate and cultured for 24 h. A total of 20 μM luciferase NF-κB and twist reporter plasmids (Apo-ONE Homogeneous caspase assay, Beyotime) were transfected into the cells using Lipofectamine 2000 (Invitrogen Life Technologies, Carlsbad, CA, USA) for 6 h. Then 2 or 5 μM piperlongumine was added, and after culturing for a further 24 h, the cells were collected. The fluorescence intensity of fluorescein was measured to determine the transcriptional activity using a dual luciferase reporter gene assay.

## Results

### Piperlongumine improves the doxorubicin sensitivity of the K562/A02 cell line

**Figure 1:**
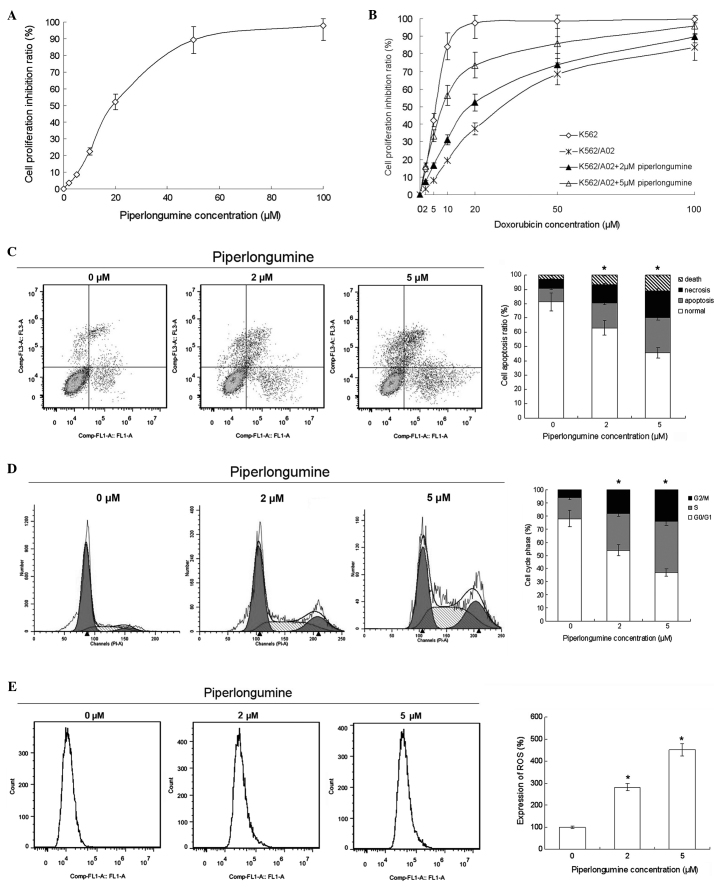
Effects of piperlongumine on the improvement of doxorubicin sensitivity in K562/A02 human leukemia cells. (A) Inhibitory effect of piperlongumine on the proliferation of K562/A02 human leukemia cells. The data are presented as the mean ± standard deviation (SD), where the bars indicate the SD; n=10. (B) Effect of piperlongumine in the reversal of doxorubicin resistance in K562/A02 human leukemia cells. The data are presented as the mean ± SD, where the bars indicate the SD; n=10. (C) Effect of piperlongumine on the apoptosis of K562/A02 human leukemia cells when subjected to doxorubicin treatment. The data are presented as the mean ± SD, where the bars indicate the SD; n=3. ^*^ P<0.05 compared with the control group. (D) Effect of piperlongumine on the cell cycle of K562/A02 human leukemia cells. The data are presented as the mean ± SD, where the bars indicate the SD, n=3, ^*^ P<0.05 compared with the control group. (E) Effect of piperlongumine on reactive oxygen species (ROS) production by K562/A02 human leukemia cells. The data are presented as the mean ± SD, where the bars indicate the SD; n=3. ^*^ P<0.05 compared with the control group.

The results of the MTS assay showed that piperlongumine suppressed K562/A02 cell proliferation with an IC_50_ of 18.3 μM. The inhibition rate of 2 μM piperlongumine was 3.5% (a non-toxic concentration), and the inhibition rate of 5 μM piperlongumine was 8.6% (a low-toxicity concentration) (Fig. 1A). Therefore, the drug-resistance reversal effect and mechanism of piperlongumine were determined at these non-toxic and low-toxicity concentrations in order to minimize interference due to toxicity issues.

With piperlongumine treatment, the sensitivity of the K562/A02 cells to doxorubicin was improved. The reversal factor, calculated as the ratio of the IC_50_ of doxorubicin in the absence of piperlongumine to that in the presence of piperlongumine was 2.8 for 2 μM piperlongumine and 6.5 for 5 μM piperlongumine (Fig. 1B). The flow cytometry results indicated that piperlongumine induced apoptosis, ROS production and cell cycle arrest in the K562/A02 cells. The cell cycle was arrested at the G2/M phase (Fig. 1C). These results suggest that piperlongumine reverses the doxorubicin-resistance of K562/A02 cells by inducing apoptosis, ROS production and cell cycle arrest.

### Piperlongumine suppresses the drug efflux of the K562/A02 cell line

**Figure 2:**
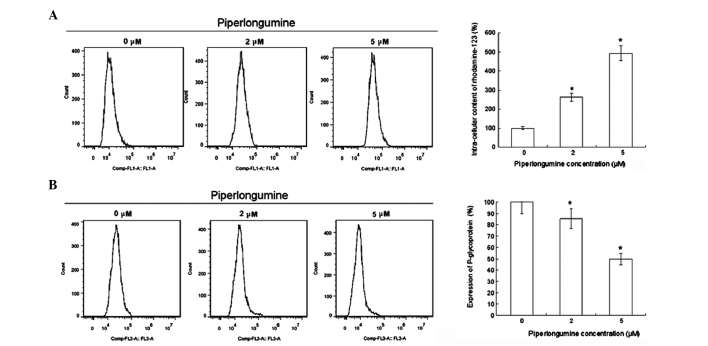
Effect of piperlongumine on the drug efflux of K562/A02 human leukemia cells. (A) The effect of piperlongumine on the intracellular concentration of rhodamine-123 in K562/A02 human leukemia cells. The data are presented as the mean ± standard deviation (SD), where the bars indicate the SD; n=3, ^*^P<0.05 vs. the control group, (B) The effect of piperlongumine on the expression of P-glycoprotein in K562/A02 human leukemia cells. The data are presented as the mean ± SD, where the bars indicate the SD; n=3, ^*^P<0.05 vs. the control group.

Efflux is an important mechanism associated with the drug-resistance of tumors (12). The intracellular concentration of rhodamine-123 was increased and the cellular surface expression of P-gp was suppressed by piperlongumine treatment in K562/A02 cells as shown in Fig. 2. These results suggest that piperlongumine also plays an important role in attenuating the drug efflux of K562/A02 cells.

### Piperlongumine regulates drug resistance-related genes and signaling pathways

**Figure 3:**
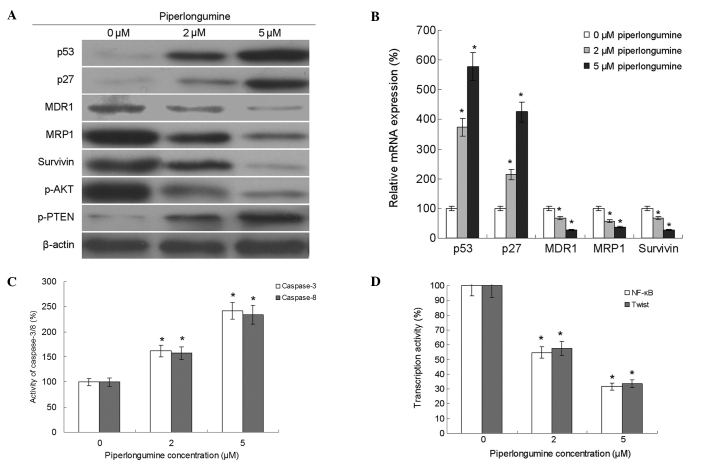
Effect of piperlongumine on drug resistance-related genes and signaling pathways of K562/A02 human leukemia cells. (A) The effect of piperlongumine on protein expression of drug resistance-related gene and signaling molecules of K562/A02 human leukemia cells. β-actin was as the internal control. (B) The effect of piperlongumine on the mRNA expression of drug resistance-related genes of K562/A02 human leukemia cells. GAPDH was the internal control. The data are presented as the mean ± standard deviation (SD), where the bars indicate the SD; n=3, ^*^ P<0.05 vs. the control group. (C) The effect of piperlongumine on the activities of caspase-3/8 of K562/A02 human leukemia cells. The data are presented as the mean ± SD, where the bars indicate the SD; n=5, ^*^ P<0.05 compared with the control group. (D) The effect of piperlongumine on the transcriptional activity of NF-κB and twist in K562/A02 human leukemia cells. The data are presented as the mean ± SD, where the bars indicate the SD; n=5. ^*^ P<0.05 vs. the control group.

The proliferation and survival of tumor cells may be increased by the regulation of drug resistance-related genes and signaling pathways (13). In the present study, the expression levels of MDR1, MRP1, survivin, p53 and p27 and the activity of caspase-3/8 were detected. The results showed that piperlongumine decreased the expression of MDR1, MRP1 and survivin, and increased the expression of p53 and p27 and the activities of caspase-3 and -8. Furthermore, the results also showed that piperlongumine decreased the phosphorylation of Akt and the transcriptional activities of NF-κB and twist. In addition, it increased the phosphorylation of PTEN (Fig. 3).

## Discussion

The development of tumor drug resistance is a multi-step, multi-stage and multi-factorial complex process. There are two major mechanisms associated with the occurrence of drug-resistant in leukemia, namely, drug efflux and drug resistance-related genes (14–19). Previous studies have shown that piperlongumine is an inhibitor of human tumors. The results of the present study demonstrate the drug resistance reversal effects of piperlongumine in K562/A02 human leukemia cells, and indicate that they are achieved by the regulation of drug efflux and drug resistance-related genes. The present study also found that piperlongumine enhanced the intracellular concentration of rhodamine-123 and reduced the cellular surface expression of P-gp, suggesting that piperlongumine may be able to suppress drug efflux. In addition, the results showed that piperlongumine enhanced ROS production by the tumor cells, suggesting that ROS may be one of triggers to drug resistance reversal.

Furthermore, the present study revealed that piperlongumine induced apoptosis and G2/M phase arrest. Enhancements in the expression levels of p53 and p27 and reductions in the expression levels of survivin and the activities of caspase-3 and -8, suggest that the effects of piperlongumine as an inhibitor of tumor proliferation and inducer of cell cycle arrest may be associated with its ability to regulate cell apoptosis and cell cycle-related genes.

Drug resistance mechanisms also involve the expression of MDR1 and MRP1 through encoding P-gp and intracellular transport proteins. They are ATP-dependent drug efflux pumps and able to actively pump lipophilic drugs out of the cells using the energy released from ATP hydrolysis due to the extremely low intracellular drug levels (20,21). The results of the present study showed that piperlongumine downregulated the expression of MDR1 and MRP, resulting in the decreased expression of P-gp and other intracellular transport proteins.

The results of the present study also showed that piperlongumine downregulated the phosphorylation of Akt, a kinase that plays a critical role in the phosphoinositide 3-kinase (PI3K) signaling pathway and regulates cell survival, differentiation, proliferation and metabolism by modulating the expression of genes such as p53 and p27 (22). Piperlongumine was also demonstrated to inhibit the transcriptional activities of NF-κB and twist in K562/A02 cells, and there is some evidence that the transcriptional activities of NF-κB and twist are associated with tumorigenesis (23–26).

In summary, this study found that piperlongumine is able to reverse the drug resistance of the K562/A02 human leukemia cell and that its mechanism of action involved the regulation of tumor drug resistance-associated gene expression. Thus, piperlongumine may potentially be useful as a new therapeutic agent for leukemia.

## Figures and Tables

**Table I:** Quantitative PCR primer sequences.

Gene	Primer sequences
p53	F: 5′-CGGTTTCCGTCTGGGCTTCTT-3′ R: 5′-CCACACGCAAATTTCCTTCCACTC-3′
p27	F: 5′-GTTAGCGGACGAGTGTCCAG-3′ R: 5′-TGTTCTGTTGGCCCTTTTGTT-3′
MDR1	F: 5′-GGAGCGGTTCTACGA-3′ R: 5′-ACGATGCCCAGGTGT-3′
MRP1	F: 5′-CACACTGAATGGCATCACCTTC-3′ R: 5′-CCTTCTCGCCAATCTCTGTCC-3′
Survivin	F: 5′-AGCCCTTTCTCAAGGACCAC-3′ R: 5′-GCACTTTCTTCGCAGTTTCC-3′
GAPDH	F: 5′-GGAGCGAGATCCCTCCAAAAT-3′ R: 5′-GGCTGTTGTCATACTTCTCATGG-3′

PCR, polymerase chain reaction; F, forward; R, reverse.
